# Grand Challenges in Sustainable and Intelligent Phytoprotection

**DOI:** 10.3389/fpls.2021.755510

**Published:** 2021-11-17

**Authors:** Kai Huang, Lei Shu

**Affiliations:** ^1^National Engineering and Technology Center for Information Agriculture, Nanjing, China; ^2^Nanjing Agricultural University, Nanjing, China; ^3^School of Engineering, University of Lincoln, Lincoln, United Kingdom

**Keywords:** smart phytoprotection, plant protection, computer science, plant science, information and communication technology

Plant enhances the capacity of the ecosystem and agricultural systems, enabling human beings to survive and multiply on the earth. Since the human being appeared, plants and their production have played a pivotal role in the development of human civilization, which is mainly reflected in four aspects, including ecological value, edible and medicinal value, ornamental value, and cultural value. In the growth process of both aquatic and terrestrial plants, we need to consider plant protection from the four following levels. 1) at the level of maintaining ecological diversity: we need to explore soil improvement and protection, ecological and supply-demand balance of precise fertilization, meteorological prediction and warning and prediction of plant pest and disease epidemic patterns, etc.; 2) at the level of agricultural production: scientific breeding and seed selection, plant phenotyping, food safety and traceability, intelligent diagnosis and control of plant diseases and pests, intelligent weeding, automatic harvesting, quality grading and division, water and fertilizer integration, quality monitoring, growth monitoring, plant nutrition maintenance, ecology, soil, fertilizer and crop resources and nutrient balance, etc.; 3) at the level of plant high standardized farmland construction: explore smart orchard, smart tea garden, smart field, smart facility farmland, etc., explore recycling agriculture, Photovoltaic Agriculture (Huang et al., 2020), green and organic agriculture; 4) at the level of sustainable development: explore low carbon models, light-weight computing, small sample models, low-cost and high-reliability equipment, open database for sharing and crowdfunding, etc. In the field of plant protection, we should not merely consider one of the above levels, but consider all, that is, from the perspective of ecosystem, landscape, and economics, which is also a grand challenge for the development of plant protection.

As a new interdisciplinary subject, Smart Phytoprotection emerges under the background of the continuous development of science and technology and the in-depth transfer from computer science to plant science. Traditional plant protection discipline mainly focuses on biological sciences which refer to other disciplines including chemistry, biology, and ecology. Then researches have been toward to reduce pesticide pollution, protect the environment, and realize ecological sustainable development.

At present, the fast development toward various information and communication technologies, e.g., Internet of Things (Chen et al., 2020), Satellite Remote Sensing (Zhang et al., 2021), Aerial Image Processing (Su et al., 2021), Big Data (Wolfert et al., 2017), Cloud Computing (Gai et al., 2020), Artificial Intelligence (Jiang et al., 2020), 5G technology (Tang et al., 2021), Blockchain (Zhou et al., 2020; Liu et al., 2021), Quantum communication (Gisin and Thew, 2007), and Robotics (Karoly et al., 2020), has provided new opportunities for agriculture applications (Friha et al., 2021), further promoting the integration of both computer science and plant science and leading to realizing “Smart Phytoprotection” with novel research ideas and solutions involved in plant protection and development, which refers to green, smart plant protection, and evolving technology. However, various challenges from different fields need to be addressed to push the boundaries of developments in Smart Phytoprotection. Specifically, due to the lack of in-depth integration between sensors, information and communication technology (ICT), and plant protection technology, some grand challenges are becoming increasingly arduous, resulting in several key research themes.

## 1. Research Field of Sustainable and Intelligent Pest Identification and Control

Plant diseases and pests pose a great threat to agriculture production, which can directly or indirectly lead to a drop of crop yield and even crop failure. It is critical to monitoring the state of plant diseases and pests efficiently and accurately, and to preventing and controlling them in time. In the agricultural field, if the identification of plant diseases, pests and weeds is carried out manually, we will face the following four main challenges:

1. There are fewer experienced farmers, and it is still difficult for new farmers to screen disease species in the early stage of the disease, or even during the outbreak;
2. Wrong or delaying diagnosis during the grow control period is the fundamental cause of large yield reduction even extinction;
3. Existing methods of pest, diseases and weed control mostly focus on chemical control, which causes large areas of soil and water pollution and is not conducive to sustainable and low-carbon development, not to mention carbon neutrality in the future. More researches should focus on physical and biological control methods;
4. Plant growers face hazards in the field and suffer from occupational diseases (e.g., lumbar muscle strain, skin disease, arthritis) caused by long-term labor work.

Similar challenges also exist while judging whether an insect is a pest or a beneficial insect, and judging what kind of a weed it is. At present, in order to effectively cope with the grand challenges, we suggest the research on the following five aspects:

1. Based on Internet of Things, Image Processing, and Knowledge Graph technology (Liu et al., 2016), we can carry out researches on intelligent diagnosis and control methods for pests, diseases and weeds of various crops. Establish intelligent diagnosis system from single disease to multi-disease and from single crop to multi-crop;
2. Based on Internet of Things, Artificial Intelligence, Satellite Remote Sensing, Aerial Image Processing, Big Data, and other technologies, we can launch the researches on intelligent prevention and control of crop, forest, and grass plant diseases, such as locust monitoring based on remote sensing;
3. Based on Internet of Things, Radar Detection Technology (Hu et al., 2016), and Drone Technology, migratory pests are monitored, prevented and controlled intelligently. For example, the use of Solar Insecticidal Lamps Internet of Things (Li et al., 2019) and radar monitoring network for insect migration;
4. Based on process-based models and statistical models, we can launch the researches on assessing the best management practices to control pests and crop diseases, and forecasting the impact of global climate change on future pest management;
5. Based on Internet of Things and Artificial Intelligence, better resources (e.g., water, pesticides) management tools and decision support system could be supplied to planters to ensure the implementation of the necessary changes (e.g., smart farming technologies).

## 2. Research Field of Green Intelligent Biological Control Technology

In the process of biological control, it is necessary to consider the impact of various factors, e.g., environmental conditions, host plants, host pests, application methods, product batches, on natural enemies of pests and pathogenic microorganisms. It is of great importance to confirm the effect of biological control through large-area monitoring. However, the traditional manual method is time-consuming and laborious to know the whole control effect accurately, which is not beneficial for the development of biological control technology, and for the establishment of evaluation standards and systems about biological control effect.

In order to effectively cope with the challenges of green intelligent biological control, we suggest the researches on the following two aspects:

1. Based on Internet of Things, Artificial Intelligence, Satellite Remote Sensing, Aerial Image Processing, Big Data, Knowledge Graph, and other technologies, we can carry out the researches on the epidemic law, and comprehensive prevention and control for plant diseases and pests, weeds, on the treatment of degraded grassland, and on the integrated soil and water management. For example, the pest monitoring network can help cultivators anticipate problems in advance and take the initiative to solve them (Wohleb et al., 2021);
2. Based on Internet of Things, Aerial Image Processing, Remote Sensing Satellites, Big Data, and other technologies, large-scale alien species invasion can be identified, monitored, prevented, and controlled, for example, the prevention and control of buffelgrass spreading in the United States (Elkind et al., 2019).

## 3. Research Field of Green Intelligent Ecological Control Technology

By intelligently adjusting the planting environment parameters, such as water, light, temperature, CO_2_, fertilizer, etc., we can create a suitable growing environment for crops, further improving the disease resistance of crops. Moreover, it is helpful to select high-quality pest-resistant varieties, improve crop planting structure, protect beneficial organisms, and control the occurrence rate of plant diseases and pests through regulating biodiversity. Therefore, it is of great significance to carry out researches on green intelligent ecological regulation technology. How to realize such green intelligent ecological control technology is a grand challenge in this research field.

At present, in order to effectively cope with the above challenges of green intelligent ecological control, we suggest the researches on the following six aspects:

1. Based on the Internet of Things, Artificial Intelligence, Satellite Remote Sensing, Aerial Image Processing, Big Data, Drones, etc., we can carry out researches on intelligent water and fertilizer irrigation integration;
2. Based on the Internet of Things, Artificial Intelligence, Intelligent Equipment, Big Data, etc., we can carry out researches on coordinating the power generation of Photovoltaic Agriculture and crop growth, intelligent control of environmental temperature, humidity, light and ventilation of facility agriculture, etc., meanwhile, focus on intelligent greenhouse of the Internet of Things;
3. Based on the Internet of Things, Artificial Intelligence, Photovoltaic Agriculture, etc., we can carry out researches on combining with light-transmitting film, multi-spectrum, automatic adjustment of light duration and CO_2_ concentration, etc., and also combining with plant nutrition, concentrate on precision organic planting;
4. Based on Internet of Things, Crowd Sensing, Aerial Image Processing, Big Data, we could carry out researches on protecting beneficial insects. For instance, prevention and control of large-scale bees' death, beekeepers' perception on environmental changes, and even pollution in bees' activity areas;
5. Based on Internet of Things, Artificial Intelligence, Satellite Remote Sensing, Aerial Image Processing, Big Data, we could carry out researches on biodiversity control for plant diseases, weeds, and pests, and on the pest prediction models by monitoring, investigation and research of pests. Then, make regulatory decisions to give full play to the natural regulatory advantages of biodiversity;
6. Based on advanced sensing, Machine Learning, and Big Data, we could use eco-evolutionary models to learn from both epidemic and genome data for deployment of disease-resistant cultivars and fungicides in space and time to both control plant diseases and impede their adaptation.

## 4. Research Field of Scientific Breeding and Quality Control Technology

Food safety is one of the most important evaluation indicators in plant protection. Due to the wide variety of varieties, it is a lack of intelligent variety recommendation to select high quality pest and disease resistant varieties. In terms of quality control technology for the whole planting process, it requires the active guarantee of each link to make the crops in food safety to be visualized and traced. Thus, it is necessary to conduct researches on the application technology of agricultural product traceability based on the Internet of Things, Artificial Intelligence, Big Data, and other technologies.

At present, in order to effectively cope with the grand challenges, we can launch the researches on the following three aspects:

1. Based on the Internet of Things, Artificial Intelligence, Satellite Remote Sensing, Aerial Image Processing, Big data, and Blockchain, we can carry out researches on variety quality resource evaluation and variety selection and breeding recommendation;
2. Based on the Internet of Things, Image Processing, Satellite Remote Sensing, Big Data, and Intelligent Equipment, we can carry out researches on crop quality grading and division techniques;
3. Based on the Internet of Things, Artificial Intelligence, GPS, Big Data, and Blockchain, we can carry out researches on agricultural product traceability and tracking control.

## 5. Research Field of Green Smart and Scientific Pesticide Application Technology

In the scientific pesticide application technology, researchers have continuously developed high-efficiency, low-toxicity, low-residue, and environment-friendly pesticides for plant diseases and pests (Ferentinos, 2018). How to improve the pesticide application level from the perspective of both technology and equipment, and to achieve intelligent, accurate, low-dosage and high-efficiency pesticide application effect, are two grand challenges for further popularization and application of the above pesticides. Thus, it is necessary to carry out scientific pesticide application technology based on Internet of Things, Artificial Intelligence, Big Data, and other technologies, e.g., intelligent spraying pesticides by unmanned aerial vehicles (Chen et al., 2021) and selective spraying with ground vehicles.

## 6. Research Field of Intelligent Weather Disaster Prevention Technology

Through analysis of current meteorological conditions, we can better adjust crops planting and management patterns. By predicting and controlling meteorological disasters, we can largely protect crops from damage and reduce crops losses. Therefore, it is of great significance to research intelligent and green technologies for preventing meteorological disasters. How to realize such technology is a grand challenge in this field. At present, in order to effectively deal with the above challenges, we can conduct researches from the following four aspects:

1. Based on the Internet of Things, Artificial Intelligence, Satellite Remote Sensing, Aerial Image Processing, Meteorological Sensors, and Blockchain, we can carry out crops' meteorological disaster analysis and early warning model research;
2. Based on the Internet of Things, Vision technology, and Machine Learning technologies, we can conduct researches on weather inspection robots. For instance, prevent and control frost disasters in tea gardens and meteorological pollution in tea gardens through moving inspections;
3. Based on the emerging LoRaWAN and other technologies, we can expand the wireless communication range of weather stations so that the agriculture and forestry environment can obtain the newest weather data in time and make weather disaster prevention work;
4. Based on Big Data Analysis, Statistics, Operations Research, and Mathematical Modeling, we can realize the estimation of the early warning and optimal response mechanism for meteorological disasters.

**Figure 1:**
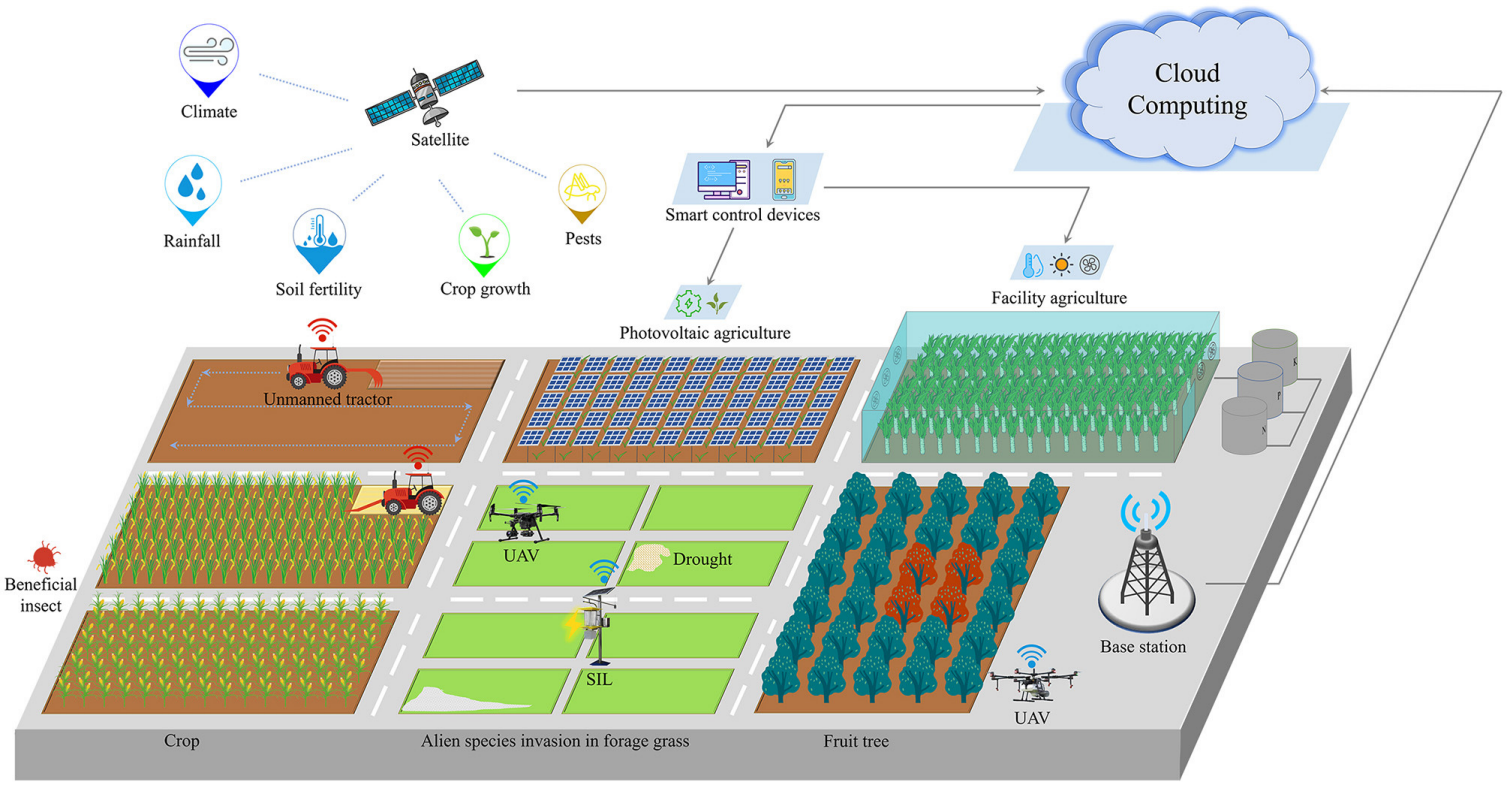
The era of unmanned smart plant protection.

In addition, while emerging cutting-edge agricultural production patterns appear, it is worth to carry out studies on new prevention and control, and protection technologies. With the continuous development of ICT, it is critical to develop and apply intelligent, compound plant protection equipment which is suitable for various agricultural production scenarios. In the future, plant protection will become sustainable, intelligent, and toward the era of unmanned smart plant protection, as depicted in Figure 1:

1. Based on Satellite Remote Sensing and Aerial Image Processing, we can carry out researches on combining with the Solar Insecticidal Lamps Internet of Things to monitor plant diseases and pests, analyzing the prevalence of plant diseases and pests, judging the effectiveness of control, and collecting climate data for weather disaster prevention;
2. Based on the Internet of Things, Artificial Intelligence Technology, Big Data, Scheduling Optimization, and UAV, we can carry out researches on accurately spraying pesticides on diseased fruit trees, intelligently reducing the amount of pesticide application and improving the effect of pesticide spraying. And the consumers can trace the pesticide application on fruit through the blockchain;
3. Based on the Internet of Things, Global Navigation Satellite System (GNSS), Intelligent Equipment, and UAV, we can carry out researches on guiding the unmanned tractor to work on the designated path and guiding the UAV to collect plant growth information on the preset path;
4. Based on Satellite Remote Sensing and Aerial Image Processing, we can carry out researches on evaluating forest and pasture germplasm resources, selecting breed, protecting beneficial insect, and monitoring the farmland, e.g., drought of farmland and the alien species invasion in forage grass.

In the era of unmanned smart plant protection, a high level of plant science is technologically achievable with the favor of computer science.

## Author Contributions

Both authors listed have made a substantial, direct and intellectual contribution to the work, and approved it for publication.

## Funding

This work was supported in part by the National Natural Science Foundation of China under (Grant 62072248), and by the Jiangsu Agriculture Science and Technology Innovation Fund under (Grant CX(21)3060).

## Conflict of Interest

The authors declare that the research was conducted in the absence of any commercial or financial relationships that could be construed as a potential conflict of interest.

## Publisher's Note

All claims expressed in this article are solely those of the authors and do not necessarily represent those of their affiliated organizations, or those of the publisher, the editors and the reviewers. Any product that may be evaluated in this article, or claim that may be made by its manufacturer, is not guaranteed or endorsed by the publisher.
